# Indolent infections in patients with hematologic malignancy: A single-center experience screening for tuberculosis and strongyloidiasis prior to cytotoxic therapy in Boston

**DOI:** 10.1016/j.lrr.2024.100489

**Published:** 2024-11-22

**Authors:** K.A. Reifler, T. Francoeur Smith, G. Bodanapu, M. Fagan, D.L. Bourque, J.M. Sloan

**Affiliations:** a Section of Infectious Diseases, Boston University Chobanian & Avedisian School of Medicine, Boston, MA, USA; b Department of Internal Medicine, Boston University Chobanian & Avedisian School of Medicine, Boston, MA, USA; c Boston University Chobanian & Avedisian School of Medicine, Boston, MA, USA; d Section of Hematology/Oncology, Boston University Chobanian & Avedisian School of Medicine, Boston, MA, USA

**Keywords:** Disseminated strongyloidiasis, Tuberculosis reactivation, Infectious screening, Pre-immunosuppression screening, Corticosteroids risk

## Abstract

Individuals with hematologic malignancy have increased risk of latent tuberculosis infection (LTBI) reactivation and *Strongyloides stercoralis* (SS) dissemination. However, screening prior to chemotherapy or corticosteroids is not routine. We conducted a LTBI and SS screening intervention amongst patients with hematologic malignancies. Over one-third immigrated from countries with high TB incidence and SS prevalence, and some had additional risk factors (HIV, HTLV-1, eosinophilia). The intervention increased screening rates but universal screening was not achieved. Screening positivity was similar pre- and post-intervention. We demonstrate a population with increased indolent infection reactivation risk. Specific infectious screening guidelines are needed to prevent significant morbidity.

## 1 Introduction

An estimated 4 % of the United States (US) population is seropositive for strongyloidiasis and 4.8 % have latent tuberculosis infection (LTBI) [1,2]. Both strongyloidiasis and tuberculosis have long indolent phases of infection that are often asymptomatic but can reactivate, particularly in the setting of immunosuppression. *Strongyloides stercoralis*, the parasite that causes strongyloidiasis, can cause asymptomatic autoinfection in an immunocompetent host for decades if untreated [3]. Infection with the bacteria *Mycobacterium tuberculosis* may be tamed by the host's innate immune response, but the organism can seed any organ and persist in granulomas for years [1]. Certain individuals are at increased risk for past exposure and/or reactivation, including those with hematologic malignancy.

In non-endemic countries, such as the US, strongyloidiasis and tuberculosis exposure are more common in individuals who migrated from outside the US, lived in congregate settings, and, for strongyloidiasis, who lived in certain parts of Appalachia or the southeastern US [1,4,5]. Individuals with acquired and iatrogenic immune system dysregulation, including those with hematologic malignancy and corticosteroid use, have increased reactivation risk [6,7]. The risk of LTBI reactivation in people with hematologic malignancy is approximately 9 times higher than in the general population, and may be higher compared to other malignancies [6]. Human immunodeficiency virus (HIV) is another important risk factor for both tuberculosis disease and strongyloidiasis reactivation [7,8]. Specific types of immunosuppression, including hematologic malignancy, chemotherapy, and human T-lymphotrophic virus type 1 (HTLV-1) infection can increase *S. stercoralis* larval production and result in hyperinfection/dissemination, which is associated with up to 90 % mortality [2,3]. Systemic corticosteroids directly accelerate transformation of the parasite into the autoinfective cycle and are associated with at least 70 % of cases of strongyloidiasis hyperinfection/dissemination, which has been observed even with short corticosteroid courses [2,3,7]. Screening for strongyloidiasis and LTBI in patients with hematologic malignancy prior to initiating chemotherapy and/or corticosteroids can identify indolent infection. Early detection may provide a window for treatment that is relatively easy and effective [4,8].

In the United States (US), screening individuals with hematologic malignancy for LTBI and strongyloidiasis is not uniformly performed and current hematology/oncology societal screening guidelines rely on hematologists’ interpretation of risk. National Comprehensive Cancer Network (NCCN) clinical practice guidelines on prevention and treatment of cancer-related infections recommend LTBI screening and state that strongyloidiasis screening may be indicated for individuals with epidemiologic risk factors and who are undergoing treatment with certain agents that are known to increase risk, such as checkpoint inhibitors [9]. During pre-hematopoietic stem cell transplant evaluation, the NCCN recommends LTBI and strongyloidiasis screening for individuals who are high risk, without defining this risk category [9]. Joint guidelines published by the American Thoracic Society (ATS)/Infectious Diseases Society of America (IDSA)/Center for Disease Control and Prevention recommend LTBI screening in individuals with high likelihood of developing active tuberculosis, including undergoing immunosuppression, and those with increased likelihood of exposure, such as migration from high-burden countries [10]. Evidence-based expert guidelines published in the Journal of the American Society of Tropical Medicine provide more specific strongyloidiasis screening recommendations [11]. They recommend screening all immunosuppressed patients and candidates for immunosuppression with high or intermediate risk of exposure (Grade 1a); risk categories are clearly defined and include exposure to specific regions, rural areas, duration of exposure, and historical exposures [11]. They also recommend screening immunocompetent individuals routinely with high-risk exposure (Grade III). However, hematologists who are often making the decision to screen individuals with hematologic malignancy may be less accustomed to consulting ATS, IDSA or tropical medicine guidelines and clear recommendations from their own professional societies are essential.

Boston Medical Center (BMC) is an urban academic medical center located in Boston, Massachusetts that serves a large immigrant population including approximately 30 % of individuals who do not speak English as their first language. Patients seen at BMC with hematologic malignancy may be at particular risk for these infections. However, predicting who will develop severe infection with these organisms is challenging and infection is not always associated with known epidemiologic risk factors [12]. For example, although >70 % of cases of active tuberculosis in the US occur in people who are foreign-born, the majority (62.3 %) of deaths from strongyloidiasis in the US occur among individuals born in the United States [5,13]. Additionally, country of birth and travel history are not always discussed in the context of chemotherapy initiation and other infection risk factors in asymptomatic patients are not always apparent. Given these challenges, we implemented a pilot intervention of universal screening for strongyloidiasis and LTBI prior to chemotherapy initiation in patients with hematologic malignancy seen at the BMC hematology clinic. The aim of this paper is to describe the results of our intervention to inform screening decisions and guidelines.

## 2 Methods

We conducted an intervention of universal screening for strongyloidiasis and LTBI for patients aged 18 years or older who were diagnosed with a hematologic malignancy and seen at BMC's hematology clinic between March 2023 and March 2024. The intervention was implemented through integration into the multiple myeloma and lymphoma electronic medical record (EMR) chemotherapy order sets and, for the remaining hematologic malignancies, screening was encouraged via educational sessions with hematology providers. Between March and October of 2023, the study team held three educational sessions for different provider groups to promote screening.

Strongyloidiasis screening was conducted by an FDA-approved IgG ELISA (Gold Stand Diagnostics) performed at a commercial laboratory for antibody to *S. stercoralis* and LTBI screening was via either QuantiFERON-TB Gold In-Tube (QFT-GIT; Qiagen; third-generation test) or QuantiFERON-TB Gold Plus (QFT-Gold Plus; Qiagen; fourth-generation test), an interferon-gamma release assay (IGRA).

Asymptomatic patients who screened positive for strongyloidiasis were treated with ivermectin 200 mcg/kg once daily for 1–2 days. Patients who screened positive for tuberculosis were referred to the BMC ambulatory tuberculosis clinic for treatment consideration.

The pre-intervention group included individuals with hematologic malignancy who were initiated on chemotherapy and seen in the clinic between December 2015 (the start of the EMR system) and March 2023. The post-intervention group included those who were seen in clinic and initiated chemotherapy during the intervention period. EMR data was extracted by the Clinical Data Warehouse. Retrospective descriptive analysis was performed including demographics, clinical characteristics, relevant lab testing, and medications. Individuals were considered to be from a high tuberculosis or strongyloidiasis endemicity region if they were born in a country whose tuberculosis incidence rate was ≥ 40 per 100,000 people or strongyloidiasis prevalence was >10 %, respectively [2,14].

Descriptive analysis was stratified by the pre- and post-intervention periods. Data are reported as trends without p-values due to the exploratory nature of the study without a specific hypothesis. Data presented in this study are deidentified and presented in aggregate and individual consent was not obtained. Research approval was obtained from the institutional review board at Boston Medical Center (H-43217) on 11/28/2022.

## 3 Results

Overall, 876 individuals were in the pre-intervention group and 83 were in the post-intervention group. Mean age was similar in the pre- and post-intervention groups (pre: 61, SD 15; post: 63, SD 14) [Table 1]. Over one-third of patients were born in a country of high TB incidence (pre: 328, 38 %, post: 27, 33 %) and nearly 40 % were born in countries with high strongyloidiasis prevalence (pre: 324, 37 %, post: 33, 40 %).

**Table 1:** Cohort demographics and medical comorbidities, stratified by intervention period.

	Pre-intervention (*n* = 876); (n,%)	Post-Intervention (*n* = 83); (n,%)
**Age in years [mean (SD)]**	61 (15)	63 (14)
**Sex (F)**	272 (42 %)	36 (43 %)
**Country of origin**		
High TB incidence⁎	328 (38 %)	27 (33 %)
Lower TB incidence⁎⁎	533 (61 %)	55 (66 %)
High strongyloidiasis seroprevalence^a^	324 (37 %)	33 (40 %)
Lower strongyloidiasis seroprevalence^b^	537 (61 %)	49 (59 %)
**Hematologic disorder type**		
Acute leukemia	69 (8 %)	12 (14 %)
Chronic leukemia	48 (5 %)	6 (7 %)
B cell lymphoma	279 (32 %)	18 (22 %)
T cell lymphoma	63 (7 %)	9 (11 %)
Plasma cell	416 (47 %)	38 (46 %)
Histiocytic & dendritic cell	1 (0.1 %)	0
**Systemic steroids**	841 (96 %)	75 (90 %)
**HIV+**	26 (3 %)	2 (2 %)
CD4 < 250	10 (1 %)	1 (1 %)
CD4 > 250	14 (2 %)	1 (1 %)
**HTLV-1+**	20 (2 %)	6 (2 %)
**Absolute eosinophilia**^c^		
Once	84 (10 %)	3 (4 %)
Two or more occasions	134 (15 %)	16 (19 %)

TB= tuberculosis; HIV = human immunodeficiency virus; HTLV-1 = human T-lymphotrophic virus type 1.

⁎ Defined as ≥ 40 cases per 100,000 people or strongyloidiasis prevalence was >10 %.

⁎⁎ Defined as ≤ 40 per 100,000 people.

a Defined as ≥ 10 % strongyloidiasis seroprevalence.

b Defined as ≤ 10 % strongyloidiasis seroprevalence.

c Defined as absolute eosinophilia count ≥ 500 per microliter.

The most common hematologic malignancy diagnosis category was plasma cell (pre: 416, 47 %; post: 38, 46 %), followed by B-cell lymphoma (pre: 279, 32 %; post: 18, 22 %), and acute leukemia (pre: 69, 8 %; post: 12, 14 %) [Table 1]. A small proportion were HIV or HTLV-1 positive (HIV: 2 % in pre- vs 3 % in post-intervention; HTLV-1: 2 %). Eosinophilia count ≥ 500 per microliter on two or more occasions was observed in 15 % and 19 % of individuals in the pre- and post-intervention groups, respectively. Over 90 % of individuals received systemic corticosteroids as part of, or in addition to, their chemotherapy regimen.

The intervention was associated with a 21 % increase in the proportion of patients screened for tuberculosis via IGRA (pre: 337, 38 % vs post: 49, 59 %) and a 41 % increase in the proportion of patients screened for strongyloidiasis via serology (pre: 159, 18 % vs post: 49, 59 %) [Tables 2 & 3]. However, universal screening was not achieved.

**Table 2:** Tuberculosis screening rates, screening positivity, and treatment.

	Pre-intervention (*n* = 876), (n, %)	Post-intervention (*n*= 83), (n, %)
**Screened for TB**^a^	337 (38 %)	49 (59 %)
IGRA Positive	73 (22 %)	9 (18 %)
Treated for Latent TB	52 (71 %)	5 (56 %)
Treated for Active TB	5 (7 %)	1 (11 %)
No treatment documented	16 (22 %)	3 (33 %)
IGRA Negative	249 (74 %)	40 (82 %)
IGRA Indeterminate	15 (4 %)	0

IGRA = interferon-gamma release assay; TB = tuberculosis.

a Screening performed via either QuantiFERON-TB Gold In-Tube [QFT-GIT; Qiagen; third-generation test] or QuantiFERON-TB Gold Plus [QFT-Gold Plus; Qiagen; fourth-generation test].

**Table 3:** Strongyloidiasis screening rates, screening positivity, and treatment.

	Pre-intervention (*n* = 876), (n, %)	Post-intervention (*n* = 83), (n, %)
Screened for Strongyloidiasis^a^	159 (18 %)	49 (59 %)
Positive Serology	4 (3 %)	2 (4 %)
Received Ivermectin⁎	7 (>100 %)	2 (100 %)

a Screening performed via commercial IgG ELISA strongyloidiasis serology.

⁎ Ivermectin 200 mcg/kg once daily for 1–2 days.

Among those screened for tuberculosis, the screening positivity rate was 22 % (*n* = 73) in the pre-intervention period. The tuberculosis screening positivity rate decreased during the intervention (pre: 73, 22 % vs post: 9, 18 %). Few patients overall had indeterminate IGRA's (15 total). Strongyloidiasis screening positivity was relatively low, including 4 (3 %) individuals pre- and 2 (4 %) post-intervention.

Among individuals with positive IGRA, the majority were diagnosed with LTBI. 71 % and 56 % of those with positive tests were treated for LTBI in the pre- and post-intervention groups, respectively [Table 2]. Six individuals total were treated for active tuberculosis (pre: 5, 7 % vs post: 1, 11 %). We were unable to find documentation of any treatment for 16 individuals in the pre-intervention and 3 individuals in the post-intervention groups. Further chart review revealed that the reasons for lack of documented EMR treatment included: therapy completion before the EMR existed, upcoming or missed appointment in the tuberculosis clinic to discuss therapy, declining therapy, and death before therapy initiation.

More patients received ivermectin (*n* = 9) than the number of individuals who screened positive by serology for strongyloidiasis (*n* = 6) [Table 3]. This is because some individuals in the pre-intervention period were treated empirically without serology testing or were treated while awaiting serology results (which can take more than a week to come back if sent out to a reference lab). No patient who screened positive for strongyloidiasis by serology was diagnosed with strongyloidiasis hyperinfection or disseminated strongyloidiasis.

## 4 Discussion

This study demonstrates a patient population with hematologic malignancy and several risk factors for reactivation of asymptomatic strongyloidiasis and latent tuberculosis infection. Over one-third of the cohort migrated from a country with high TB incidence and nearly 40 % migrated from countries with high strongyloidiasis prevalence, highlighting the importance of asking about migration history prior to starting steroids or chemotherapy. Almost every patient received steroids in addition to chemotherapy, and some had additional risk factors including HIV, HTLV-1, and eosinophilia.

The intervention increased screening rates for both strongyloidiasis and tuberculosis, but, despite our intention, universal screening was not achieved. Our results must be interpreted within the context of this bias, and individuals with a higher pre-test probability of infection may have been more likely to receive screening, particularly in the pre-intervention period. We believe universal screening was not achieved because screening was not integrated into all the chemotherapy EMR care plans and sometimes required manual provider ordering. This underscores the difficulty for providers to remember to screen for asymptomatic infections, particularly when professional society guidelines lack clear guidance.

The 18–22 % LTBI screening positivity rate in this cohort was significantly higher compared to the US national estimate of 4.8 %, although within the range of prior LTBI screening programs in individuals with hematologic malignancy outside the US [13,15,16]. This is perhaps unsurprising given the large immigrant patient population seen at BMC. Treating individuals who are starting chemotherapy for LTBI can be challenging given the drug-drug interactions and/or possible hepatoxicity. Some individuals in our cohort declined therapy due to these concerns, and others had to start chemotherapy imminently before being able to see a tuberculosis provider. Despite these challenges, IGRAs may be more sensitive before immunosuppression given the possibility of anergy, and if identified early, there may still be a treatment opportunity window [8]. Further studies are needed to understand how tuberculosis disease incidence varies across hematologic malignancies to help prioritize screening interventions.

Our cohort had increased risk factors for strongyloidiasis exposure and reactivation. However, the strongyloidiasis 3–4 % screening positivity rate was similar to the estimated US national strongyloidiasis prevalence of approximately 4 %, and lower than strongyloidiasis seroprevalence estimates for migrants from endemic countries living in non-endemic countries (approximately 12 % according to a meta-analysis performed in 2019) [2,4]. This discrepancy may be related to the limitation of the screening test, which is approximately 89 % sensitive and is thought to be reduced in those who are immunocompromised [3]. Yet serology remains the recommended screening method and screening organ transplant donors and recipients has been found to save lives, and is not always dependent on known epidemiological risk factors [3,5,12]. Although conclusive recommendation for universal screening in people with hematologic malignancies cannot be made based on this small dataset, it is worth consideration particularly with high-risk patient cohorts similar to our patient population.

No patient in this cohort was treated for strongyloidiasis hyperinfection/dissemination, perhaps due to screening and/or presumptive treatment. Given the possibility of false-negative strongyloidiasis testing in this cohort, and the fact that treatment with ivermectin is generally well-tolerated, presumptive treatment for individuals at risk regardless of serology is another approach considered by some experts [11]. A European study found that empiric ivermectin therapy without serology was cost-saving for a general population of migrants from strongyloidiasis endemic countries who were living in Europe [17]. Their model has important differences from our cohort because they assumed an immunosuppression prevalence of 2.7 % and only treated migrants from endemic countries presumptively. Our cohort had higher reactivation risk given 100 % were immunosuppressed and included individuals born in the US who may also have exposure risks. Given the morbidity of hyperinfection and disseminated strongyloidiasis in people who are immunocompromised, and the fact that treatment with ivermectin is generally well tolerated, presumptive treatment is another approach to consider. Cost-effectiveness analyses in the US are needed to understand the potential impact of universal screening compared to empiric therapy in the US.

These results highlight the need for increased attention towards pre-immunosuppression infectious screening recommendations in individuals with hematologic malignancy, particularly for infections like tuberculosis and strongyloidiasis with long latent states. We demonstrate that guidelines exist but are not necessarily in forums that are familiar to the providers making the screening decisions. We also demonstrate that achieving universal screening is challenging. Future efforts to achieve this and study efficacy would likely require integration into every chemotherapy EMR order set. Additional studies are needed to clearly define individuals’ exposure and reactivation risk factors and compare approaches such as screening versus presumptive therapy in high-risk individuals. Future guidelines should be easily interpretable and accessible to hematologists to increase prevention of these infections with public health implications and significant morbidity.

## CRediT authorship contribution statement

**K.A. Reifler:** Writing – review & editing, Writing – original draft, Formal analysis, Data curation, Conceptualization. **T. Francoeur Smith:** Writing – review & editing, Writing – original draft, Methodology, Funding acquisition, Formal analysis, Data curation, Conceptualization. **G. Bodanapu:** Writing – original draft, Formal analysis. **M. Fagan:** Writing – review & editing, Methodology. **D.L. Bourque:** Writing – review & editing, Supervision, Methodology, Conceptualization. **J.M. Sloan:** Writing – review & editing, Supervision, Resources, Methodology, Conceptualization.

## Declaration of competing interest

The authors declare that they have no known competing financial interests or personal relationships that could have appeared to influence the work reported in this paper.
